# Knowledge and Attitudes toward Cornea Donation among Different Social Groups in Poland

**DOI:** 10.3390/jcm10215031

**Published:** 2021-10-28

**Authors:** Dominika Szkodny, Ewa Wróblewska-Czajka, Edward Wylęgała

**Affiliations:** 1 Chair and Clinical Department of Ophthalmology, Faculty of Medical Sciences, Zabrze Medical University of Silesia, 40-760 Katowice, Poland; ewroblewska-czajka@sum.edu.pl (E.W.-C.); ewylegala@sum.edu.pl (E.W.); 2 Department of Ophthalmology, District Railway Hospital, 40-760 Katowice, Poland

**Keywords:** corneal transplant, donation, awareness, tissue procurement

## Abstract

Background: Limited access to corneal tissue for transplantation remains a challenge in many parts of the world. To date, little attention has been paid to the problem of the cornea donor shortage in Poland, where the number of waiting patients exceeds the number of transplants performed three-fold. The aim of this study was to assess the knowledge and willingness towards participating in corneal donation among different social groups in Poland. Methods: This prospective, cross-sectional study was conducted among health professionals, medical students, clerics, teachers, journalists, employees and patients of the District Railway Hospital in Katowice. Online and paper questionnaires were used to collect socio-demographic data and information regarding awareness of, knowledge about and attitudes toward corneal donation. For health professionals and medical students, the questionnaires contained additional questions concerning knowledge and solutions for expanding the donor pool. Descriptive analysis and associations were evaluated using the chi^2^ test. Results: In the survey, 1026 participants took part, including 370 (36.06%, group 1) health professionals and 656 (63.94%, group 2) participants from a non-medical field. A total of 330 (89.18%) from group 1 and 528 (80.49%) respondents from group 2 expressed willingness to donate their corneas. The main reason for refusal of donation in both groups was a lack of knowledge concerning eye donation (7.8%). A social campaign (64.6%) was the most frequently chosen solution for increasing the number of potential donors by health professionals and medical students. In the group of doctors, not knowing how to report a potential donor was chosen as the greatest source of difficulty in donor reporting (40%). Conclusions: In the present study, the willingness to donate one’s eyes was substantial in both groups. Social campaigns and improving knowledge concerning the donor reporting process among health professionals might be beneficial in expanding the donor pool.

## 1. Introduction

Corneal transplantation is a highly effective procedure in treating multiple corneal disorders, with great potential to improve visual acuity. However, its performance is limited by the availability of corneal tissue from deceased donors. The most important problem to be solved in transplantology is the shortage of donors, and thus the shortage of organs and tissues in relation to the number of potential recipients. The drastic disproportion between the supply and demand of donor corneas worldwide, in which there are 70 patients per one acquired cornea, has been reported in a recent global survey of eye banking and corneal transplantation [1]. In affluent countries, such as Singapore, France, Germany, Australia, Italy, and the United Kingdom, with high-quality healthcare services and an efficient eye-banking system, annual rates of corneal transplants per capita are comparable and estimated in the range of 55 to 75 × 10^−6^. In contrast, Poland, also being a developed country, is distinguished by an exceptionally low keratoplasty rate of 32 × 10^−6^. Limited access to graft tissue remains a challenge in many parts of the world, with 32 countries not performing corneal transplants and 31 with a corneal transplant rate below 5 × 10^−6^, mainly in Asia and Africa [2]. Limited access to graft tissue remains a challenge in many parts of the world, including Poland. Acquiring corneas in Poland, which is a country of 38 million people, is extremely ineffective. In Poland, about 1000 corneas are transplanted annually, of which over 500 come from multi-organ donations. Currently, 2971 patients are waiting for a corneal transplant in Poland, while the number of deaths last year was 477,335. This trend is similar to previous years, resulting in three patients waiting for one cornea. Eye banks are the institutions responsible for collecting, processing and distributing donated eye tissue for transplants, helping to narrow the gap between supply and demand. At present, in Poland, there are seven eye banks involved in corneal tissue procurement. However, donor reporting remains at a very low level, creating a constant shortage in transplant tissue. Most of the research is devoted to factors limiting multi-organ donation; much less is known about the reasons for refusing to donate one’s cornea. This is even more important when taking into account the fact that the donation of corneas has a consent rate significantly lower than other organs [2]. Different studies have established certain factors influencing the donation rate, such as religious aspects, the awareness and education of society, the presence of a presumed consent system, the number of transplant programs or organizations available in a country, family factors and others [3]. In contrast to Germany, in Poland, there is no electronic database of family interviews conducted by hospital staff regarding donation, and therefore the exact approval rate of corneal donation is impossible to establish [2]. There are two main systems around the world for becoming a donor—an “opt-in” system, where cells, tissues or organs may be removed from a deceased person if the person had expressly consented to such removal during his or her lifetime, and an “opt-out” system, valid in Poland, which permits material to be removed from the body of a deceased person for transplantation unless the person had expressed his or her opposition before death. There is a continued dispute whether the opt-out or the opt-in system has the most advantages in terms of increasing donor rate. In Poland, presumed consent is in force, which means that the removal of cells, tissues or organs from a human corpse for transplantation may be performed if the deceased person has not objected during his lifetime and family objection has no legal force. However, doctors usually ask family for permission to retrieve tissues or organs from their relative and respect their will. Contrary to the situation in our country, Spain, where the form of consent is also presumed, is described as an example for a successful donation system, with 40 deceased donors per million population [4]. Different studies present contradictory results regarding the impact of these two systems on donation rate. Countries with the highest donation rate, such as the USA, using an opt-in system, and Portugal, Belgium, and Spain, using an opt-out system, have covered the demand for tissues despite the various forms of consent [4,5]. Nevertheless, the matter of organ and donor procurement is complex and different aspects, such as sociocultural attitudes, public health institutions, the economy, the organization of the health service and other factors, should be considered. To the best of our knowledge, so far, no research has been conducted in order to explain this problem in Eastern Europe. The main aim of this project was to identify factors influencing ineffective corneal procurement by assessing willingness and knowledge regarding corneal donation in different social groups in Poland.

## 2. Materials and Methods

This descriptive cross-sectional survey followed the principles of the Declaration of Helsinki. In order to assess the awareness and attitude of various social groups toward eye donation, two questionnaires were drawn up—one for medics and a second for other groups (Supplementary materials). 

Therefore, two major groups were included—medical and non-medical. In the healthcare professionals group, doctors, nurses and paramedics from three local hospitals were enrolled. Only doctors from the anesthesia, neurology, internal medicine and surgical departments were enrolled in the study, as they represent potential professionals reporting donors. To extend the group, other health professionals were also asked to fill out the questionnaire via e-mail. The student group consisted of those having lectures in the Ophthalmology Department, District Railway Hospital in Katowice. The non-medical group included patients admitted to the hospital for COVID-19 vaccination, teachers from local schools, journalists and priests asked to fill out the questionnaire personally or through e-mail. 

The questionnaire for healthcare professionals consisted of questions regarding demographic data, including sex, age and profession, one about the source of knowledge about corneal transplants, nine questions concerning the process of corneal procurement, all single-choice and closed, four questions connected with willingness to donate corneal tissue, and one regarding solutions for improving the situation, which was a multiple-choice question with pre-set answers. The second questionnaire contained, apart from demographic data, such as sex, age, educational level and profession, three questions concerning knowledge about eye donation and five connected with willingness to donate one’s corneas. In both questionnaires, questions regarding reasons for donation refusal included the option other, with the opportunity to write their own answer.

Respondents under 18 years old and who did not consent to participate were excluded from the study. 

Informed consent was obtained from all participants. The study was conducted between January 2020 and May 2021. Throughout this period, questionnaires were successively collected. Healthcare professionals were the first group studied, and questionnaires from this group were collected until August 2020. In the second half of 2020, teachers, journalists and priests were enrolled in the study. With the beginning of COVID-19 vaccinations in our hospital, patients scheduled for vaccination were asked to fill out the paper questionnaire. Participants taking part in the online survey required approximately two or three recalls to fill the questionnaire, while paper forms were mostly filled out after one recall. 

## 3. Statistical Analysis

Data from paper questionnaires as well as online surveys were collected and compiled and analyzed in Excel spreadsheets (Microsoft, Redmond, WA, USA) separately for the medical and non-medical groups. Data are presented as the percentage of people indicating a given response for qualitative variables and the mean and standard deviation for quantitative variables. The chi^2^ test was used to compare qualitative variables between groups, while quantitative variables were compared using Student’s t-test in the case of two groups or the analysis of variance (ANOVA) with Tukey’s post hoc test for more than two groups. *p* values < 0.05 were considered significant. The statistics were prepared using MS Excel and R language in the Rstudio environment (RStudio Team (2020). RStudio: IntegratedDevelopment for R. RStudio, PBC, Boston, MA URL http://www.rstudio.com/, accessed on 1 September 2021).

## 4. Results

The test power calculated using the G power software for the chi^2^ test with the numbers above 300 was 0.99. 

### 4.1. Demographic Data

This study consisted of 1026 participants—370 health professionals and 656 participants from a non-medical field. A total of 446 healthcare professionals were contacted, which resulted in an 83% response rate. Approximately 90 doctors from local hospitals were excluded, due to them belonging to departments other than anesthesiology, surgery, and internal medicine, including cardiology and neurology. All respondents from the other groups were enrolled in the study. In the non-medical group, 1986 participants were contacted, providing a 51.66% response rate. In the first group, the most populous age group was in range of 18–30 years (59.73%), in which 22.16% of respondents were males and 77.84% were females. In this group, 34.32% of participants were nurses, 30.27% were doctors, 28.11% were students and 7.3% were paramedics (Table 1). The largest number of respondents in the second group was found in the age range of 30–50 years (39.69%). This group included 41.22% males and 58.78% females. Higher education was declared by 61.07% participants, and secondary and basic education by 28.24% and 10.68%, respectively (Table 2). The main professions in this group, apart from other (51.6%), included clergy (22.29%), teachers (7.63%) and journalists (3.66%). There were the statistically significant differences between the two groups both in terms of demographic data (sex, age and educational level) and the answers to the questions provided (*p* < 0.000).

### 4.2. Health Professionals Group

**Figure 1:**
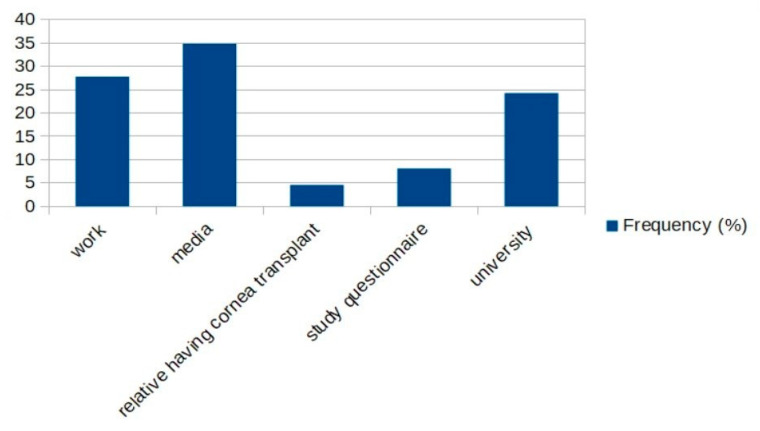
The source of knowledge regarding corneal transplants in the first group.

Media was the main source of knowledge (34.87%), followed by university (24.32%) and the workplace (17.84%). Only 5.14% of medics had ever reported a potential donor, and only 26.22% were aware of the low reporting rate of donors in Poland (Figure 1).

Not knowing how to report a potential donor was the most frequent obstacle chosen by respondents (13.51%), with a lack of time occupying second place (4.05%). Most of the health professionals (78.65%) had knowledge regarding the current form of consent in Poland; however, as many as 21.35% were not aware. In this group, willingness to donate was very strong—90.54% of participants would consent to being a donor after death, and 91.08% would not have an objection to a corneal retrieval from their close relatives. The main reason for donation refusal, including from a family member, was the lack of knowledge regarding eye donation (3.24% for themselves and 2.70% for relatives). The greatest number of people opposed to consenting to a transplant was found in the 30–50 and 50–70 age groups. There seems to be a tendency towards a reluctance to donate one’s cornea that increases with age (*p* = 0.137). Paramedics and nurses declared their willingness to donate cornea less frequently than other groups (*p* = 0.026). The level of knowledge showed a positive correlation with willingness to be a donor (Table 3 and Table 4). Doctors had a significantly higher level of knowledge than the other groups (*p* < 0.000), and the other professions did not differ significantly.

**Figure 2:**
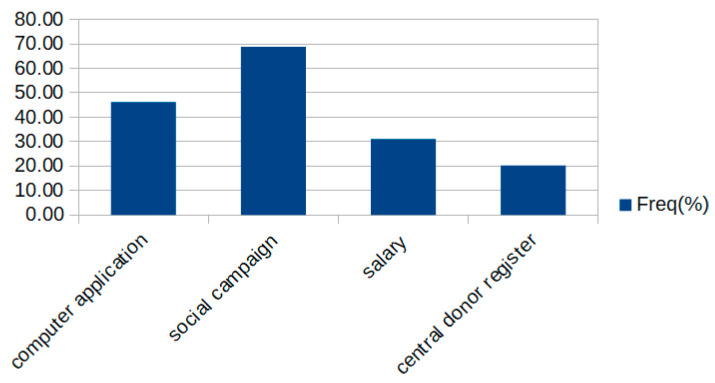
The most effective solutions for improving the situation in Poland.

As for the most effective solutions for improving the situation in Poland regarding the shortage of potential donors, participants chose a social campaign (68.92%) and a computer application facilitating donor reporting (46.22%) (Figure 2). In the 30–50 and 50–70 age groups, most people chose a higher salary for reporting doctors (*p* = 0.104).

### 4.3. Non-Medical Group

**Figure 3:**
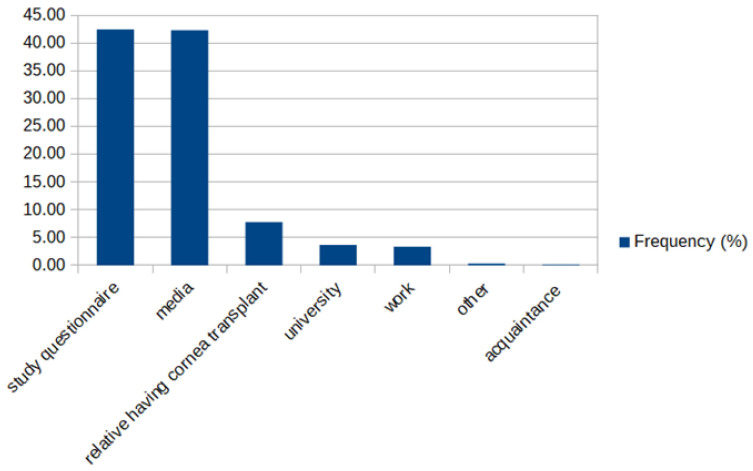
The source of knowledge regarding corneal transplants in the second group.

A substantial number of participants in the second group had never heard about corneal donation, and the questionnaire was the first source regarding this issue (42.44%) (Figure 3).

**Figure 4:**
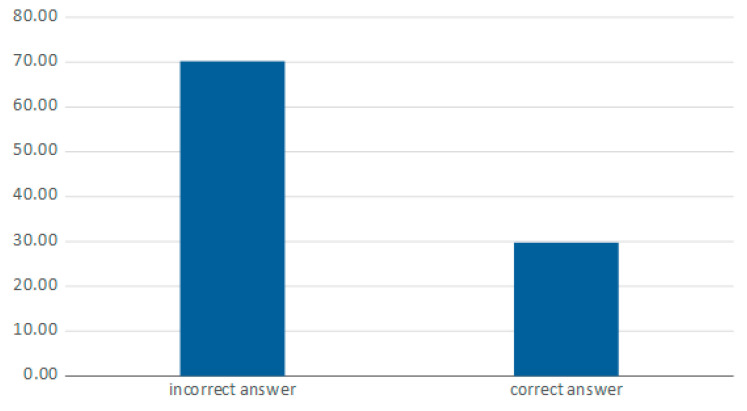
The form of consent being in force in Poland.

The vast majority of respondents did not have knowledge about the form of consent in force in Poland (70.23%) (Figure 4).

Additionally, in this group, 58.32% of participants were unaware about the cornea donor age limit, and 50.84% did not know that cornea donation is only possible after death. Regardless of the poor awareness, willingness to donate corneal tissue in the second group was also strong (80.61%); however, only 59.39% of respondents would have signed up for the central donor register, if there was one. At the same time, slightly fewer respondents were not against donation from their relatives (73.59%). Similar to the first group, a lack of knowledge was the main reason behind refusing donation (10.23% and 9.31% for relatives) (Table 5).

The decreasing tendency regarding the willingness to become a cornea donor with age was also observed in this group (*p* = 0.000), as well as consenting to obtain corneas from relatives (*p* = 0.008) and registering as a cornea donor (*p* = 0.005) (Table 6). 

The highest number of correct answers regarding the form of consent in Poland and the possibility of retrieving corneas from a living donor was observed among respondents with higher education (*p* = 0.000 and *p* = 0.000, respectively). The greatest reluctance to become a potential donor (*p* = 0.000), to consent to a cornea donation from a family member (*p* = 0.000) and to show willingness to sign up to a donor registry (*p* = 0.000) was observed in the group of people with basic education. The majority of incorrect answers regarding the form of consent was observed among people who gave their friends (*p* = 0.000) or the survey (*p* = 0.000) as a source of knowledge. People pointing to the media responded slightly better, as well as people who knew someone after a transplant, people who obtained this knowledge during their studies and who drew from other sources of knowledge. In the question about donor age limit, most of the correct answers were indicated by people who knew someone after a corneal transplant (*p* = 0.000). Slightly fewer correct answers were given by people declaring their university education and the media as sources of knowledge. The worst results were those indicating other sources of knowledge. Belonging to a certain professional group had no association with willingness to donate one’s corneas.

## 5. Discussion

The study presents the awareness of and attitudes toward corneal donation among two major groups, a medical and a non-medical group, comparable to the study performed by A. Dave et al. [6] and, contrary to some other surveys, including only one kind of social group [7,8]. However, a significant number of respondents took part in the survey (1026), far exceeding the number in similar studies, such as in Ghana (100 participants), North India (507), Pondicherry, India (196), North Ethiopia (774) and Japan (371) [6,7,8,9,10]. As in those studies, sociodemographic data, knowledge regarding corneal donation and reasons for refusal to become a donor are assessed. Despite the persistently low donor reporting rate in Poland, in our study, willingness to donate corneal tissue was high both in the group of health professionals (90.54%) and non-medical group (80.61%). In comparison, in the studies from other countries, the willingness to become a cornea donor reached 68.24% in North India, 67.3% in Ghana and 37.6% in Ethiopia [6,7,8,10]. The study disclosed several factors limiting willingness to become a donor, such as the age of participants, degree of education and lack of awareness regarding eye donation. A lack of adequate knowledge and awareness about eye donation as well as a lower level education were also identified in other studies as elements affecting donation consent [6,7,8]. Our study included two groups—health professionals, to verify aspects influencing reporting potential donors, and people from outside the medical community, to search for factors having an impact on consent rate. To the best of our knowledge, ours is the first study assessing the knowledge and attitudes regarding eye donation in Poland.

Since Poland is an 88% Christian country and donation issues used to arouse ethical and religious controversy [10], in our study, a substantial part of the study group included monks and priests. The level of willingness to donate one’s cornea was comparable among the clerics compared to other participants (*p* > 0.005); therefore, it seems that religious issues are not a barrier to donation in Poland [8]. In the study conducted by Michalska et al., 81% of clerics would sign a declaration of intent and 79% of respondents declared that they would not be in opposition in the case of a donation from a dead relative, which was similar to other students outside the medical school [9]. Additionally, we observed no correlation between belonging to a given professional group and eye donation consent, including teachers and journalists. In the previous studies, specific unwillingness toward corneal donation was reported [11,12]. According to a 2016 Center for Assessment of Public Opinion (CBOS) survey, 93% of Poles approve of transplantation [13]. The consent rate for eye donation in our study was slightly lower than in the CBOS report, but was comparable to this score in both groups (89.18% and 80.49%), and therefore it is reasonable to assume that there is no specific reluctance toward cornea donation in Poland.

There is also a general dispute regarding whether the type of consent has an impact on the effectiveness of organ and tissue procurement. In a global survey conducted by Gain P et al., it was described that an opt-out system promotes donation [10]. The concept of presumed consent is being used in Polish transplantation law, but active objections can be voiced by close relatives. The awareness regarding the type of consent in force in Poland was very low in the non-medical group (29.77% of correct answers) and not satisfactory in the group of health professionals (78.65%). Substantially fewer respondents would have signed up to a donor registry, if it existed, than were willing to donate their corneas (59.39% vs. 80.61%). The opt-in system makes the conversations with family easier, because the will of the deceased is known; however, it potentially limits the donor pool, as shown in our study. In the study performed by A. Arshad et al., no significant differences were observed in the number of deceased donors, comparing the situation of opt-in countries with opt-out countries. The United States, with a 30.7 per million deceased donation rate, have implemented an opt-in system, while Belgium, Portugal and Spain, applying an opt-out system, also have a donation rate of over 30 per million. The situation is analogous to the countries with a low donation ratio—Mexico with an opt-in system and Greece with an opt-out system [4]. Taking into account the fact that certain countries have succeeded in overcoming the donor shortages using different consent systems, it seems that other areas are crucial in the organ and tissue procurement process.

Limited knowledge and limited awareness were revealed to be contributing factors for donation refusal in our study, and are cited as the only modifiable factors that may change the attitude toward this issue [14,15] The main source of knowledge of non-medical respondents who had heard about corneal transplant before was the media, which is similar to the study performed by S. Lartey et al. [8]. Therefore, it is reasonable to assume that social campaigns could increase awareness and willingness to donate corneal tissue. Furthermore, in a study conducted by Tsigkos D. et al., it was shown that a 5 min interactive online survey can have a significant impact on changing the mentality towards organ donation [14]. These findings display the fact that rising awareness in society may increase the donation consent rate and efforts should be maintained to provide detailed information about eye donation to the families of deceased [16]. Nevertheless, our study revealed that approval rate for cornea donation, either from respondents themselves and from their family member, was high (80.61% and 73.59%). Considering these results, it is quite likely that difficulty in obtaining consent is not the only and probably not the main reason for the shortage of donors in Poland.

Other reasons for organ and tissue scarcity have been discussed, such as non-recognition of a potential donor, which depends on a system between different hospitals and the eye banks [17,18]. It is suggested that an improved eye donation coordination network could enhance the eye donation rate, including a comprehensive review of all hospital deaths and the use of a well-defined protocol [3]. In Poland, the eligibility criteria for cornea donors are in accordance with the European Eye Bank Association guidelines and are consistent with the Eye Bank Association of America guidelines [19]. These guidelines also include completing a Donor Risk Assessment Interview, concerning, among other things, homosexuality, which is considered to be a contraindication to retrieve corneas in many countries, including Poland. However, taking into account the development of modern screening tests and the results of studies identifying the number of potentially lost donors, reconsidering donor eligibility criteria such as the death-to-explantation interval and male homosexuality could also positively affect tissue procurement, which is taken into account in some parts of the USA [20,21]. 

The time after death to cornea procurement in Poland is limited to 24 h; however, for example, in Germany, this period is extended to 72 h. There are also differences regarding cornea procurement from oncology patients even between hospitals in Poland, where some hospitals collect tissue from such patients and others do not. This also narrows the donor pool, while the only definitive contraindications according to EEBA include retinoblastoma, hematological neoplasm, and malignant tumors of the anterior segment of the eye. Finally, only 5.14% of surveyed health professionals have ever reported a cornea donor, and as the main problem in this process, respondents indicated a lack of knowledge regarding this procedure. From the solutions proposed in the responses to the questionnaire for increasing the donor pool, respondents most frequently chose social campaigns and a computer application facilitating donor reporting, while a salary increase for those reporting donors was preferred by almost one-third of respondents.

This study has several limitations. First of all, despite the large number of overall participants, the studied group was heterogeneous in terms of profession and educational level, which may influence the generalization of these results and presents a sample of willingness to donate corneas in Poland. Secondly, the two studied groups differed in terms of demographic data, however the aim of the study was not to compare both groups but to assess the attitude towards cornea donation separately for medical and non-medical respondents, assuming that healthcare professionals have more knowledge on this topic. Furthermore, most of the participants were from the Silesia region, and may not exactly reflect the situation in different parts of our country. Finally, the response rate in the second group was relatively low, and therefore the opinion of those who were not willing to take part in the study is not known, and it is possible that they have a more negative attitude toward cornea donation than the participants.

To conclude, the results of the study indicate that the willingness to donate corneas in Poland is significant; however, raising awareness in the Polish population through social campaigns could have a positive effect on increasing the donation consent rate. Work should be undertaken to improve and facilitate donor reporting, including an incentive system and innovative tools such as computer applications to make this process more effective.

## Figures and Tables

**Table 1:** Distribution of socio-demographic characteristics of the first group.

Gender	Frequency (%)
Male	22.16
Female	77.84
**A** **ge**	
18–30	59.73
30–50	26.22
50–70	13.51
>70	0.54
**Professional group**	
Doctor	30.27
Paramedic	7.30
Medical student	28.11
Nurse	34.3

**Table 2:** Distribution of socio-demographic characteristics of the second group.

Gender	Frequency (%)
Male	41.22
Female	58.78
**A** **ge**	
18–30	20.46
30–50	39.69
50–70	34.96
>70	4.89
**Professional group**	
Primary school	10.68
High school	28.24
University	61.0

**Table 3:** Association between correct answers to questions regarding knowledge about corneal donation and willingness to donate. The column entitled ‘Average correct answers’ presents the number of correct answers on questions about corneal donation. Knowledge was measured by the number of correct answers.

	Number of Participants	Average Correct Answers on Questions Regarding Knowledge about Corneal Donation	Standard Deviation	*p*-Value
Yes	336	3.36	1.41	0.001
No	34	2.50	1.48	
**Would you give your consent to have a cornea collected from a family member?**				
Yes	338	3.34	1.41	0.007
No	32	2.63	1.60	

**Table 4:** Association between willingness to donate corneal tissue and socio-demographic data in health professionals.

**Would You Consent to Being a Cornea Donor?**					
	**Yes**		**No**		
**Age**	**Number of Participants**	**Percentage of Participants**	**Number of Participants**	**Percentage of Participants**	***p*-Value**
18–30	209	62%	12	35%	*p* = 0.014
30–50	84	25%	13	38%	
50–70	41	12%	9	26%	
>70	2	1%	0	0%	
**Would You Consent to Being a Cornea Donor?**					
	**Yes**		**No**		
**Professional Group**	**Number of Participants**	**Percentage of Participants**	**Number of Participants**	**Percentage of Participants**	***p*-Value**
doctor	106	19%	6	31%	*p* = 0.026
paramedic	23	13%	4	7%	
students	100	13%	4	30%	
nurse	109	56%	18	32%	

**Table 5:** Reasons for donation refusal.

Reason for Donation Refusal	Frequency (%)
not applicable	80.31
lack of knowledge	10.23
body disfigurement	2.29
opinion of relatives	2.29
religious reasons	1.68
other reason	2.91

**Table 6:** Association between willingness to donate cornea tissue, age and education.

**Would You Consent to Being a Cornea Donor?**		
**Age**	**Frequency (%)**	
18–30	20	*p* = 0.000
30–50	20	
50–70	50	
>70	10	
**Education**		
primary school	6	*p* = 0.000
high school	28	
university	67	
**Would You Give Your Consent to Have a Cornea Collected from a Family Member?**		
**Age**	**Frequency (%)**	
18–30	20	*p* = 0.008
30–50	20	
50–70	50	
>70	10	
**Education**		
primary school	5	*p* = 0.000
high school	28	
university	68	

## Data Availability

Data available on request due to privacy and ethical considerations.

## Supplementary Materials

The following are available online at https://www.mdpi.com/article/10.3390/jcm10215031/s1, Questionnaire Groups.
