# Supplementary material for: Knowledge and Attitudes toward Cornea Donation among Different Social Groups in Poland
Source: J Clin Med. 2021 Oct 28;10(21):5031. doi: 10.3390/jcm10215031 (PMC8584702; doi:10.3390/jcm10215031)
Supplement: Supplementary file 1 [file jcm-10-05031-s001.zip › jcm-1366526-supplementary.pdf]

Questionnaire Groups

Questionnaire Group 1:

1. Gender:
   - □ female
   - □ male
2. Age range:
   - □ 18–30
   - □ 30–50
   - □ 50–70
   - □ >70
3. Profession:
   - □ doctor
   - □ paramedic
   - □ medical student
   - □ nurse
4. Where does your knowledge of corneal transplantation come from?
   - □ media
   - □ university
   - □ work
   - □ I, or someone around me, had a corneal transplant
   - □ this is the first time I encounter this issue from the current survey
5. Have you ever reported a potential donor?
   - □ yes
   - □ no
6. What is the situation of corneal transplants in Poland in terms of number of performed keratoplasties?
   - □ very good
   - □ moderate
   - □ very bad
   - □ don' know
7. What has the greatest impact on reducing the number of corneal transplants performed?
   - □ lack of qualified personnel
   - □ lack of appropriate equipment
   - □ not enough tissue for transplantation
   - □ don't know
8. Where the cornea tissue from the deceased person may be retrieved?
   - □ in the operating room during multiorgan donation
   - □ in the dissecting room

□ both answers are correct
□ don’t know

9. What is the biggest obstacle for you in reporting a potential donor?
□ not knowing how to report a donor
□ talking to the donor's family
□ lack of time at work
□ the issue of low financing the process of donor reporting
□ I am not entitled to report a donor

10. Is there an upper age limit for cornea donors?
□ yes
□ no

11. At what time after death should the corneas be collected?
□ up to 2 h
□ up to 6 h
□ up to 24 h
□ don’t know

12. What is a contraindication to cornea retrieval?
□ HIV, hepatitis B, hepatitis C, syphilis, encephalitis
□ glaucoma, cataract
□ any chronic disease
□ don’t know

13. What form of consent to be a donor is valid in Poland?
□ presumed consent
□ consent registered in the Central Register of Consents
□ handwritten consent
□ don’t know

14. Would you consent to being a cornea donor after death?
□ yes
□ no

15. For what reason would you refuse to be a cornea donor?
□ religious issues
□ due to the aesthetics of the body after death
□ because of my relatives' views on the subject
□ due to a lack of knowledge about corneal transplants
□ inny powód – jeśli to możliwe proszę podać jaki
□ not applicable

16. Would you give consent to have a cornea collected from a close relative after his / her death?

□ yes

□ no

17. Why would you not consent to a cornea being collected from a close relative after his / her death?

□ religious issues

□ due to the aesthetics of the body after death

□ because of my relatives' views on the subject

□ due to a lack of knowledge about corneal transplants

□ inny powód – jeśli to możliwe proszę podać jaki

□ not applicable

18. How do you think the situation regarding cornea transplantation in Poland could be improved? You may choose more than one answer

□ increasing the remuneration for persons involved in reporting and collecting tissues

□ conducting a social campaign on cornea transplantation

□ facilitating reporting by introducing an appropriate computer application

□ introducing the Central Register of Consents and not Objections

Questionnaire Group 2:

1. Gender:

□ female

□ male

2. Age range:

□ 18–30

□ 30–50

□ 50–70

□ >70

3. Education

□ primary school

□ high school

□ university

4. Profession

□ teachers

□ clergy

□ journalists

□ other

5. Profession

□ teachers

□ clergy

□ journalists

□ other

6. Where does your knowledge of corneal transplantation come from?

□ media

□ university

□ I, or someone around me, had a corneal transplant

□ this is the first time I encounter this issue from the current survey

7. Is there an upper age limit for cornea donors?

□ yes

□ no

8. What form of consent to be a donor is valid in Poland?

□ presumed consent

□ consent registered in the Central Register of Consents

□ handwritten consent

□ don't know

9. Can you only donate a cornea after death?

□ yes

□ no

10. Would you consent to being a cornea donor after death?

□ yes

□ no

11. For what reason would you refuse to be a cornea donor?

□ religious issues

□ due to the aesthetics of the body after death

□ because of my relatives' views on the subject

□ due to a lack of knowledge about corneal transplants

□ inny powód – jeśli to możliwe proszę podać jaki

□ not applicable

12. Would you give consent to have a cornea collected from a close relative after his / her death?

□ yes

□ no

13. Why would you not consent to a cornea being collected from a close relative after his / her death?

□ religious issues

□ due to the aesthetics of the body after death

□ because of my relatives' views on the subject

□ due to a lack of knowledge about corneal transplants

□ inny powód – jeśli to możliwe proszę podać jaki

□ not applicable

14. Would you register as a potential cornea donor, if there was an appropriate registry?

□ yes

□ no
